# From the name to the popular image of the plant: the Polish names for the black elder (*Sambucus nigra*)

**DOI:** 10.1186/s13002-024-00649-0

**Published:** 2024-01-30

**Authors:** Olga Kielak

**Affiliations:** Faculty of Languages, Literatures and Cultures, Maria Curie-Skłodowska University in Lublin, plac Marii Curie-Skłodowskiej 4a, 20-031 Lublin, Poland

**Keywords:** Ethnolinguistics, Ethnobotany, Plant names, Black elder (*Sambucus nigra*)

## Abstract

The names of plants convey information on their appearance (shape, structure, colour), taste or smell, their uses (practical, ceremonial, magical and medicinal) as well as the beliefs and convictions associated with them. Assuming that the particular features of plants, entrenched in their names, must have been important to language users for some reason, the analysis of plant names can help reconstruct traditional knowledge about plants. The author analyses the standard and dialectal names for the black elder (*Sambucus nigra*) in Polish, juxtaposing the plant’s features revealed in its names (linguistic data) with the cultural accounts associated with the plant (“with-linguistic” data). This allows for the reconstruction of the following features of the plant: (a) the appearance of the shrub, (b) the properties of its fruit, (c) the smell of the plant, (d) the place where it grows, (e) the time of harvesting, (f) its use in folk medicine and (g) the association of the plant with impure powers and diseases. The conducted analyses show that reaching for hard “linguistic evidence” (standard and folk names) makes it possible to compile hierarchies of the characteristics of the plants described. Situating these names against the background of “with-linguistic” data leads to the conclusion that folk nomenclature and folk knowledge enrich and complement each other. The vast number of names for the black elder with different onomasiological bases, presenting different points of view, also demonstrate the relationship between the degree of lexical differentiation and the cultural meaning of the plant. The ethnolinguistic analysis of the names for the black elder (*Sambucus nigra*), similarly to ethnobotanical studies of folk plant names, provides insights into past and contemporary uses of the plant. Thus, it can provide a starting point for further ethnobotanical research.

## Introduction

The study of folk names of plants, often pursued by linguists, constitutes a rather old branch of ethnobotany which gives insights into the past and contemporary uses of plants. Phytonyms are a source of traditional knowledge about plant biodiversity, which, as a result of social change and economic development, is gradually disappearing. Thus, issues related to the collection and analysis of popular names of individual plants are gaining importance [1].

Authors of ethnobotanical publications sometimes collate only the popular names of selected plants [2, 3] or attempt to associate the name of a plant with its characteristics [cf. 4–6]. The latter approach is much closer to my practice. The works listed above, in some limited scope, are reminiscent of ethnolinguistic publications whose authors analyse the names of individual plants with a view to reconstructing their linguo-cultural images [7–11].

In cognitively oriented linguistics, it is believed that naming the world is, in a sense, tantamount to taming, domesticating it. Names, especially the names of plants that interest me, are not accidental—they convey information on the plant’s appearance (shape, structure, colour), taste or smell, but also other perceptible properties, such as sharpness, stickiness or clinginess; there are also names motivated by the use of specific plants or the beliefs and convictions associated with them [cf. 12–19]. The particular features of plants must have been important to language users for some reason if these were eventually incorporated into their names. Thus, the analysis of phytonyms makes it possible to discover or reconstruct the traditional knowledge about plants.

Issues concerning the perception, naming, classification and use of plants, their interaction with other species, their harvesting, processing and use, as well as aspects concerning cultural knowledge of plants fall not only within the research field of ethnobotany [20], but also that of ethnolinguistics [21]. Ethnolinguists, similarly to ethnobotanists, are fascinated by the world of plants, in particular they are interested in studying the relationships holding between the plant world and humans. Both ethnolinguistics and ethnobotany focus on the exploration of popular thinking; both disciplines also share a special treatment of language, which, as it turns out, is not only natural for linguists who, upon constructing descriptions of plants, reach for hard “linguistic evidence” that allows them to verify many of the hypotheses they put forward, but also for ethnobotanists, whose research often prioritises aspects of knowledge encoded in language [21].

Based on the assumption that scholars undertaking ethnobotanical research do not necessarily have to be naturalists by education, since ethnobotany is indeed a multidisciplinary field of research [20], the analysis of the popular names of the black elder (*Sambucus nigra*) presented in this article also provides the possibility to test the applicability of linguistic methodology to ethnobotanical research.

This approach—in which ethnobotany and ethnolinguistics (or broader: linguistics) meet—is not new. Cognitive and linguistic research was popular in ethnobotany in the 1960s and 1970s—the analysis of lexemes was undertaken by, *inter alia*, Brent Berlin, Dennis Breedlove and Peter Raven [22], Robert Randall and Eugene S. Hunn [23] or Cecil Brown [24, 25]. Nowadays, this approach is successfully applied by the Russian researcher Valerija Kolosova [26–29], who treats ethnobotany as a part of ethnolinguistics—the field of science that examines language through the lens of human consciousness.

Although I am familiar with the folk taxonomy of plants presented by Brent Berlin [30], I am much more interested in issues related to naming—the multitude of names with different onomasiological bases used in Poland to describe a single plant. The subject of this analysis is the standard and dialectal names of the black elder (*Sambucus nigra*). The choice of this plant was not accidental—in Polish folklore (and if one were to look further, also in the folklore of other Slavic peoples, e.g. Russians, Ukrainians and Belarussians [31] or in Germanic beliefs [32]), it is regarded as an untouchable plant, both sacred and cursed at the same time. It is associated with the good and the evil sacred, and was commonly used in folk medicine and magic. The folk names of the plant reflect many of the characteristics of this shrub present in ethnographic accounts. The article also provides a broader cultural context for the collected names, in accordance with the approach adopted by the Lublin-based community of ethnolinguists, following the postulate of holistic description, i.e. a combined analysis of linguistic, textual (although these are poorly represented in the article) and “with-linguistic” (including folk beliefs and practices) data [33]. In this article, I would like to show how, based on an ethnolinguistic analysis of the dialectal names of the black elder (*Sambucus nigra*), it is possible to reconstruct the folk image of this plant, to isolate many of the essential characteristics of the shrub (which are later enriched and supplemented by data of a different type).

## Methods and sources

The contexts containing the aforementioned folk beliefs, convictions and practices were extracted from ethnographic sources published at the turn of the nineteenth and twentieth centuries (monographs and articles published in ethnographic journals, such as “Zbiór Wiadomości do Antropologii Krajowej”, “Wisła” or “Lud”), as well as from published field recordings of folk accounts.

The above procedure, i.e. the analysis of strictly linguistic data against the background of “with-linguistic” data, or, to put it differently, the juxtaposition of the characteristics of the black elder revealed in the names (linguistic data) with cultural accounts associated with the plant (“with-linguistic” data), makes it possible to reveal the motivation behind the dialectal names of the black elder, and thus, to reconstruct selected fragments of knowledge about the plant.

The above-mentioned selected fragments of the linguo-cultural image of the black elder will be reconstructed by means of the cognitive definition proposed by Jerzy Bartmiński, the founder of Lublin-based ethnolinguistics. The main goal of the cognitive definition “is to capture the way an object is understood by speakers of a given language, i.e. the way that is socially entrenched, derives from knowledge about the world, the categorisation of its phenomena, their characteristics and evaluation, all of which can be accessed through language and language use” [34]. In cognitive definitions, stereotypical judgements that give rise to images of the defined objects are arranged into semantic subcategories called facets. The individual facets that make up the cognitive definitions of plants include issues related to the plant’s naming, cultivation, location; its practical, ritual, magical and medicinal use; the plant’s role in annual and family customs and rituals; its symbolism (e.g. a cognitive definition of myrtle (*Myrtus communis*) reconstructed using the cognitive definition method, see [21].

The semantic subcategories, together with their content, are not mechanically included by the authors in the definitions of the individual entries, but reconstructed for each of the described plants in such a way as to bring out, through them, the hidden characteristics of a particular plant in the analysed material from the point of view of the bearer and participant of the studied culture [35]. Although different subcategories will appear for each plant, emerging from the collected documentary material, in principle a certain set will reappear. Selected subcategories, most frequently recurring in the plant descriptions, are summarised in Table 1.

**Table 1:** The semantic subcategories, together with their content

Complexes and collections	With which things and phenomena does the described plant co-occur, performing a common function
Oppositions	What are the oppositions typical of the plant and what feature becomes the basis for juxtapositions
Origin	What is the origin of the plant
Appearance and characteristics	The appearance of the defined plant (size, colour of flowers or fruit) and its characteristics (e.g. scent)
Flowering and harvest time	The flowering period of the plant and the time of harvesting its fruit
Cultivation and care	Information relating to the cultivation of the described plant
Location	Place of occurrence (growth) of the plant
Locator	Who lives/resides in the plant
Blessing	When and under what circumstances the plant is blessed
Practical use	Information on all the practical uses of the plant
Ritual use	Information on the use of the plant in annual rituals (e.g. Christmas, Easter, St. John’s Day) and family rituals (christening, wedding and funeral)
Magical use	Information on the use of the plant in magic (for protection, love, household)
Medicinal use	Information on the use of the plant in folk medicine and in folk veterinary medicine; both in medically justified practices and in magical treatments
Prophecies + divinations	How the plant is used by man to infer the future
Equivalents	A list of “cultural substitutes” for the plant, i.e. objects that appear in the same functions
Symbolism	This concludes the explication part, summarises the definition of the plant

In cognitive definitions of plants, the distinguished semantic categories are preceded by two segments of the definition that are not facets—the segment referred to as “names”, which presents the dialectal names of the given plant and etymological issues, and the segment “categorisations”, which contains a compilation of the higher-order concepts for the plant being defined, which later determine the further selection of semantic categories [cf. 21].

Before proceeding with the analysis proper, one point requires further specification. In colloquial Polish, the name *bez* refers to two different bushes—*czarny bez* [black elder] (*Sambucus nigra*) and *bez lilak* [lilac] (*Syringa vulgaris*), also known as *lilak pospolity* [common lilac]. Although the name *bez* was originally used to designate *Sambucus nigra*, in the eighteenth century the name was transferred to another ornamental shrub imported to Poland, *bez lilak* (*Syringa vulgaris*) [36], “probably due to its scent, as both species of shrub are characterised by an intense but dissimilar fragrance” [37].

The nineteenth- and twentieth-century ethnographic literature most often refers simply to *bez*, with ethnographers rarely specifying which species of *bez* (the elder or the lilac) they were actually referring to. According to some folklore scholars, “all this folklore does not really refer to the lilac (*Syringa vulgaris*), commonly called *bez*, but only to two species of the genus *Sambucus*” [38], i.e. the black elder (*Sambucus nigra*) and the dwarf elder (*Sambucus ebulus)*; and further: “the lilac, as a recently introduced shrub, has no folklore in our country, and it only shares with the elder the name, which was transferred to it” [38]. Despite their similar names in Polish, the two species have a different image and symbolism in folklore, as shown by the author of the dictionary entries for *czarny bez* [the black elder] and *bez* (*lilak*) [the lilac] published in Issue 7 of the volume on plants of the ethnolinguistic *Słownik stereotypów i symboli ludowych* [*Dictionary of Folk Stereotypes and Symbols*] [39, 40].

## Results and discussion

In Polish, the black elder (*Sambucus nigra*) is called simply ***bez***, in dialects it is also referred to as ***best***, ***bezd***, ***bezg***, ***besz***, ***bestek***, ***bziak***, ***baźnik***, ***bzówka*** [41], ***bzianka*** or ***bzica*** [42].

The general Slavic name *bez* (cf. Czech *bez*, Russian and Ukrainian *бeз*, Bulgarian *bъz,* Serbian and Croatian *bazga/baza* [36] is related to Latvian *buzga* “stick” [43] and Lithuanian *bezdas*, *bezas*, *bezdis*, *bezdus* “black elder” [38].

Some researchers, such as the Polish archaeologist and ethnographer Erazm Majewski, derived the name of the black elder (*Sambucus nigra*) from the unpleasant smell emitted by the shrub [38]. Similarly, according to the etymologist Aleksander Brückner, “the source of the name could be a strong smell related to *bdźda* [digestive gas]” [44]. Polish villagers associated the name of the shrub—*bez*—with the similar-sounding word *bies* [fiend, demon, devil], which in Polish folklore means an evil spirit; according to the ethnographer Franciszek Kotula, in the Rzeszów region the word *bez* was pronounced “in a whisper and with fear in the eyes”; the initiated anxiously argued that *bez* meant the same as *bies* [45]. Both characteristics of the elder entrenched in the name will be referred to again later in this article.

The standard Polish and dialectal names of *Sambucus nigra* entrench the appearance of the shrub: the dark colour of the fruit: ***czarny bez*** [black elder] (common) and the whiteness of the flowers: ***dziki biały bez*** [wild white elder] [41], ***biały bez*** [white elder] [42], ***biały best*** [white elder] [46]—in contrast to the purple colour of the flowers of the lilac (*Syringa vulgaris*), called *lilak* [lilac] (common), *liliowy bez* [*lilac elder*], *bez siwy* [grey elder] “with lilac-coloured flowers”, *bez siwasy* [*greyish lilac*] [41], *bez niebieski* [blue elder] [47] or *modry bez* [deep blue elder] [41]. In the dialects of the Żywiec region, the black elder is called ***baranie jaja*** [ram’s eggs/balls] due to the resemblance of the plant’s fruit to the testicles of that animal [48].

The characteristic unpleasant odour of the plant, from which—according to the aforementioned etymological hypothesis—the name black elder is derived, is also entrenched in numerous dialectal names: ***bez śmierdzący*** [stinking elder] [41], ***best śmierdzący*** [stinking elder] [49], ***śmierdziel*** [stinker] [42]; the black elder is sometimes called ***smrodzina***, ***smrodynia***, ***trzemcha*** or ***kocierbina***—these names were transferred to *Sambucus nigra* from the bird cherry (*Prunus padus*) due to the perceived similarities between the two shrubs, i.e. white, unpleasantly smelling flowers and black fruit [48].

The characteristics of the plant encapsulated in the name are confirmed by ethnographic data—in “collections of superstitions about plants” published in ethnographic journals from the turn of the nineteenth and twentieth centuries, there are frequent references to the fact that the black elder emits a strong smell, its flowers “give a strong and not very pleasant smell” [38]. This view is shared by contemporary rural inhabitants of the Lublin region, who say of the black elder that *te kwiaty śmierdzo, to krzak mocno śmierdzący* [*the flowers stink, it is a strongly smelling shrub*] [42]. An attempt to explain the unpleasant (cadaverous!) smell of the black elder can be found in an aetiological legend, according to which the leaves, flowers and fruit of the plant have a cadaverous smell since Judas hung himself on it [50].

The black elder (*Sambucus nigra*), called ***dziki bez*** [wild elder], ***leśny bez*** [forest elder] [41], ***bez polny*** [field elder] [51], grows wild in forests and thickets [52], in ditches [53], along field roads and balks [54], in the field [55]—in contrast to the lilac (*Syringa vulgaris*), called *bez ogrodowy* [garden lilac], which grows near the house [41]. According to a colloquial account recorded in the Lublin region: *to, co dziko rośnie, to jest czarny bez, a przy domu to bez* [*what grows wild is the black elder, and near the house it is lilac*] [42]. The location of the black elder entrenched in the names has its justification in folk beliefs. It was believed that nothing would grow in a place where a black elder bush was cut [55], and that it would be haunted [56]. In some areas of Poland, it was believed that no houses should be erected where the black elder used to grow [52], “because there will often be ailments” [57].

The association of the plant with impure powers is present in the names of the black elder; not only were the two similar-sounding words *bez* and *bies* [45] associated with each other, but also—for instance, in the Lublin region—*Sambucus nigra* was called ***diaboli bez*** [devil’s elder] [58]. This association is strongly established in Polish folk culture. It used to be believed that the shrub was planted by the devil [59], and for this reason, it is sometimes referred to as *diabelskie nasienie* [devil’s seed] [56]. In folk imagery, the elder bush is inhabited by a number of demonic beings: an evil spirit [56], the devil [60], a witch [61]; for this reason, its fruit was not harvested in Eastern Poland [59], and children were warned not to “toss” its branches for fear of attracting “some misfortune” [62].

At the same time, in Pomerania and Kashubia, as well as in Greater Poland, it was believed that also good spirits resided under the black elder shrub: dwarfs [63], household spirits [64] and benevolent demons looking after the farmyard where the black elder grows [65]. However, the association with “the good sacred” was not entrenched in the names of the plant.

The black elder was also called ***gościec*** [rheumatoid arthritis] [66]—the name has to do with beliefs according to which diseases and paralysis reside in the elder [56]; in the shrub or under its roots resides plica [67] and rheumatoid arthritis [55]. In Ropczyce, it was believed that rheumatoid arthritis resides in the black elder and whoever cuts the shrubs will break their arms and legs [55] and will suffer from rheumatoid arthritis [68]. Bone pains and plica was believed to result from snapping or breaking branches of the black elder at the wrong time of the year [69], or even accidentally by moving the plant’s roots [67].

The ban on cutting down the black elder also had a practical reason; cf. the recorded colloquial account: *jak u kogoś rósł, to nie wycinali, bo miał gotowe lekarstwo*; *czymu wycinać bez, kiej ludzi leczy?* [*when the black elder grew at someone’s place, they did not cut it down because they had a ready remedy; why cut down the elder if it cures people?*] [56].

Although, as a general rule, the black elder was not supposed to be dug up, cut or broken, in certain circumstances it was permissible to do so. In the Lublin region it was believed that the black elder could only be dug up for medicine, in the Poznań region—for a cure against plica [70]. For medicinal purposes, elderflowers were harvested at a specific time, namely on the eve of St John (23 June) and because of that the plant was called ***świętojanny best*** [St John’s elder] [48]; the elderflowers picked at this time were kept at home and used throughout the year [71]. It was not advisable to pick elderflowers after St John’s Day, as it was believed to be contaminated by witches (*cioty je oszczały* [hags urinated on them]) [56].

The black elder, called ***bez lekarski*** [medical elder] [52], ***bez apteczny*** [pharmaceutical elder] [65], has commonly been used in folk medicine. The elderberry juice or jam [56] or tea made from elderflowers [72] was used to treat the common cold. Black elder blossoms were considered to have antipyretic and diaphoretic properties, hence elderflower infusion was used to relieve fever [73]. In the past and in modern times, elderberry has been used to treat the cough that accompanies a cold, cf. in a colloquial account: *Czarny bez od kaszlu to było już obowiązkowe. To miało być jako lek* [*The black elder for coughing was obligatory. It was used as a cure*] [58]. Dried elderflowers were used for asthma, to treat shortness of breath [56], the plant was used to treat hypertension [72], heart and lung diseases [56], angina, pharyngitis and stomatitis [65]. Black elder blossoms and fruits were used for upset stomach; they were used to treat intestinal ulceration and bladder disorders [56]. The plant was commonly used for various skin ailments, e.g. fresh elder leaves were used to cover pimples [65]; consumption of elderberry jam was recommended for the measles and pox [74]; fresh, grated elder leaves were applied to burned areas [75]. For more on the medicinal properties of the black elder [see 39, 76].

*Sambucus nigra* was also called ***bez podlewany*** [watered elder] “the black elder poured with herbal water, in which a sick person was bathed” [41], and the name reveals the once common practice of pouring water under the shrub, e.g. after washing a patient suffering from rheumatoid arthritis [66], or water after washing sick children, “so that the *evil* residing under the elder takes away child’s illness” [38].

The black elder was also referred to as ***psi bez*** [41], ***psi best*** [46] or ***psiuch*** [dog’s elder] [42]—these names may be linked to the fact that raw elderberries are inedible [77]; they are suitable for eating in a processed form—in the form of jam [78], marmalades and juice [52], wine [79] or soup: the so-called *bzówki* [41] or *bzianki* [56]. Due to the fact that dog did not enjoy high reputation among the villagers (“everything among the people, when talking about something bad or unpleasant, is similar to a dog: bad, thin, hard etc. as a dog; barks (talks a lot) as a dog; finally: cold, hungry, hot, dark, it rains as a dog, and even: it hurts as a dog” [80]), the attribute “dog’s” was usually used to refer to inferior species of plants that were of no particular use to humans—inedible, unpalatable, etc. [81].

The work that an ethnolinguist performs when elaborating a cognitive definition of a plant consists in a detailed analysis of the collected material and in “filtering out” from it the characteristics of the plant; the individual characteristics (stereotypical judgements about the plant) are arranged into semantic subcategories/segments of the definition (Table 2).

**Table 2:** Documentary material and a characteristic of the described plant

Documentary material	A characteristic of the described plant that has been filtered out and assigned to a semantic category/segment of the definition
[Ethnographic description] “The black elder was not only terrible in Bieśnik, it could be found everywhere. In some areas, it was also called the “watered elder” since herbal water, in which a man suffering from rheumatism was bathed, was poured under the elder shrubs”. [45]	Water, in which a sick person suffering from rheumatism was bathed, was poured under the black elder → medicinal use The black elder was called the watered elder → names The black elder was categorised as a shrub → categorisations
[Legend] “When Judas betrayed the Lord [Jesus] and then doubted himself, he decided that he would hang himself. However, he could not find a suitable tree for this, for they were all too tall. Then he saw a black elder shrub growing by the roadside and hanged himself on it. By doing so, he stigmatised the plant, and the stigma is still a reminder of the unfortunate event. The leaves, flowers and fruit of this shrub have a corpse-like smell”. [50]	Judas hung himself on the black elder → locator The black elder has an unpleasant (corpse-like) smell since Judas hung himself on it → the appearance and properties of the black elder are categorised as a shrub → categorisations
[Ethnographic description] “On this [St John’s] feast day they harvest elderberries against sweats, and this elderberry they dry for the whole year”. [71]	The elderberry was harvested on St. John’s eve → harvest time; Dried elderberries were used as a sweat-inducing remedy → medicinal use
[Ethnographic description] “The cut tangle was tied in a rag together with a few cents and put in a hollow in a tree or in holes in the church wall, or thrown into wild black elder shrubs (called *gościec*—rheumatism), on crossroads […] and sometimes buried in the ground”. [66]	The cut tangle was thrown into black elder shrubs → medicinal use The black elder was called *gościec* → names The black elder is categorised as a shrub → categorisations
[Popular account] “The stinky elderberry is a cure for various diseases, the berries used to be picked and dried”. [42]	The black elder has an unpleasant smell → appearance and properties The black elder was called stinky elderberry → names Dried elderberries were used as medicine → medicinal use

Although, as it has already been mentioned, a full cognitive definition is created based on three types of data: linguistic, textual and “with-linguistic” data; a partial reconstruction of the linguistically entrenched representation of a plant can be made based on just one type of data. The aforementioned linguistic evidence (names for the black elder) allows for “certification of characteristics”, i.e. a certain selection of characteristics that can be used to reconstruct certain segments of the linguistic worldview [cf. 82].

The names of the black elder (*Sambucus nigra*) analysed in this article allow us to reconstruct the following characteristics of the plant: (a) the appearance of the shrub (colour of the flowers, colour and shape of the fruit), (b) characteristics of the fruit (raw—inedible), (c) the characteristic unpleasant smell of the plant, (d) the place where it grows (in forests, along field roads), (e) the time of harvesting, (f) the way it is used in folk medicine—both in practices considered to be medically justified and in practices of a magical nature and (g) the association of the shrub with impure powers and diseases (Table 3).

**Table 3:** The characteristics of the black elder entrenched in its names

Characteristics of the plant	Names
Appearance of the shrub: Flower colour Fruit colour Fruit shape	*dziki biały bez*, *biały bez*, *biały best* *czarny bez* *baranie jaja*
Characteristics of the fruit	*psi bez*, *psi best*, *psiuch*
Smell	*bez*, *bez śmierdzący*, *best śmierdzący*, *śmierdziel*, *smrodzina*, *smrodynia*, *trzemcha*, *kocierbina*
Place of growth	*dziki bez*, *leśny bez*, *bez polny*
Harvest time	*świętojanny best*
Medicinal use	*bez lekarski*, *bez apteczny*,* bez podlewany*
The association of the plant with impure forces and diseases	*bez*, *diabli bez*, *gościec*

The characteristics of the black elder entrenched in its names influence the arrangement and shape of the categories that make up the reconstructed image of this plant. A full cognitive definition of the black elder, reconstructed on the basis of a complete set of data (linguistic, textual and “with-linguistic” data), published in the form of an entry in *Słownik stereotypów i symboli ludowych* [*Dictionary of Folk Stereotypes and Symbols*] [39], consists of the following semantic subcategories (facets): origin; appearance and properties; time of flowering and harvesting; location; locator; prohibition of digging up, cutting and breaking the black elder; blessing; practical, ceremonial, magical, medicinal uses; divination; prophecy; equivalence; symbolism. The distinguished semantic subcategories are preceded by two segments of definition that are not facets: names and categorisations. Most of the facets listed are already revealed upon analysing the linguistic data, i.e. the names of the black elder.

Reaching for hard “linguistic evidence” (general Polish and popular names in folklore) allows ethnolinguists reconstructing the imagery of individual plants to hierarchise the characteristics of the object according to the assumption that the names preserve the most significant features of that object from the human perspective. At the same time, the analysis of names against the background of “with-linguistic” data (folk beliefs and practices, but also folk accounts), i.e. a holistic, comprehensive description, which enables capturing the onomasiological mechanisms, shows that folk nomenclature and folk knowledge enrich and complement each other.

The multitude of names for the black elder (*Sambucus nigra*) with different onomasiological bases, representing different points of view: perceptual—highlighting certain physical features of the plant, functional—related to the function performed by the plant, or cultural—based on thinking about the plant in a socio-communicative context [83] confirms the observations made by B. Berlin, D. Breedlove and P. Raven concerning the positive correlation between the degree of lexical differentiation and the cultural meaning of the plant [22].

Of course, the black elder is no exception in this respect. Similar naming patterns can also be observed in relation to other plants (not only shrubs, but also flowers or herbs), the folk reconstructions of which have been published in individual plant-centred volumes of *Słownik stereotypów i symboli ludowych* [*Dictionary of Folk Stereotypes and Symbols*]. These include, for instance, *głóg* [hawthorn] (*Crataegus*)—a thorny plant (*ciernie*, *bodlak*, *kolidupa*) with white flowers (*ciernie białe*) and red flowers (*kogutki*), floury fruits (*kulasza*, *babia mąka*), eagerly eaten by birds (*głóg ptasi*, *ptasi chleb*), used in folk medicine (*babiorka*, *babicha*) and associated with an evil spirit (*diabla gruszka*) [84]; *kaczeniec* [marsh-marigold] (*Caltha palustris*)—a plant with yellow, small flowers resembling small ducklings (*kaczeniak*, *kacze ziele*, *żłótacz*), growing in marshlands (*bagno*, *błotuch*), blooming at the end of April, around St Adalbert’s Day (*wojciech*), and used to treat, inter alia, colic (*kolkownik*) [85]; or *przestęp* [white bryony] (*Bryonia alba*)—the Polish equivalent of the mandrake (*mandrygula*, *nedregula*) known in many cultures of the world and associated with the devil (*ziele djable*, *diabelska rzepa*), a plant with yellowish-white roots and flowers (*białe ziele*, *biała macica*), which was used to treat ulcers (*uśpiwrzód*) [86].

## Conclusion

The ethnolinguistic analysis of phytonyms, similarly to the ethnobotanical studies of folk plant names, gives insight into ancient (sometimes already forgotten) and contemporary uses of plants. It can thus be a starting point for further ethnobotanical research.

## Data Availability

The research data that is the basis for the publication (lexicographic and ethnographic material) will be made available in the Zenodo repository.
